# tACS suppresses M1 polarization of microglia after spinal cord injury through the cGMP-PKG signaling pathway

**DOI:** 10.3389/fimmu.2026.1907391

**Published:** 2026-10-07

**Authors:** Haidi Bi, Ke Huang, Xiaojun Zhou

**Affiliations:** 1 School of Public Health, Jiangxi Medical College, Nanchang University, Nanchang, China; 2 Jiangxi Provincial Key Laboratory of Disease Prevention and Public Health, Nanchang University, Nanchang, China; 3 Department of Rehabilitation Medicine, the 1st affiliated hospital, Jiangxi Medical College, Nanchang University, Nanchang, China; 4 The 1st Clinical Medical College School, Jiangxi Medical College, Nanchang University, Nanchang, China

**Keywords:** cGMP-PKG, microglial polarization, neuroinflammation, spinal cord injury repair, TACs

## Abstract

**Background:**

Strategies for repairing spinal cord injury (SCI) emphasize blocking the shift of microglia to the pro-inflammatory M1 phenotype, lowering neuroinflammation, and fostering neuronal survival. The cGMP-PKG pathway modulates immune cell function by decreasing the secretion of pro-inflammatory elements such as IL-1β and TNF and boosting the levels of the anti-inflammatory element IL-10, thereby alleviating inflammatory responses. Additionally, transcranial alternating current stimulation (tACS) at specific frequencies may indirectly regulate immune cell activity and inflammatory cytokine secretion by modulating the autonomic nervous system, thereby demonstrating anti-inflammatory potential.

**Objective:**

To assess how tACS influences microglial polarization following SCI and investigate the underlying molecular mechanisms.

**Methods:**

Proteomics sequencing, Western blotting, ELISA, Nissl staining, hematoxylin and eosin (HE) staining, real-time quantitative PCR (qPCR), immunofluorescence, live/dead staining and other experimental techniques were employed. Furthermore, an *in vivo* rat model was used to determine the effect of tACS on the recovery of motor function following injury.

**Results:**

The polarization of glial cells towards the M1 proinflammatory phenotype was partially suppressed by tACS, thereby alleviating neuroinflammation and improving neuronal survival to promote functional recovery after SCI. Mechanistic studies revealed that tACS modulates the conversion of the glial inflammatory phenotype by initiating the cGMP-PKG signaling pathway, thereby offering neuroprotective and anti-inflammatory effects.

**Conclusion:**

The activity of the cGMP-PKG signaling pathway is upregulated by tACS, thereby modulating microglial polarization, suppressing neuroinflammation and protecting neurons from inflammatory injury. As a non-invasive neuromodulation technique, tACS holds potential as a therapeutic approach for SCI.

## 1 Introduction

Spinal cord injury (SCI) frequently results in irreversible sensory and motor dysfunction (1–3). Its prognosis is dictated by both primary mechanical trauma and subsequent secondary damage, which involves a mutually amplifying cascade of inflammation, oxidative stress, excitotoxicity, and blood-spinal cord barrier disruption (4–6). These processes collectively exacerbate neuronal loss and impede axonal regeneration and circuit reformation, making precise management of the post-SCI neuroinflammatory microenvironment critical for functional recovery (7–9).

Microglia, the resident immune cells of the central nervous system, dynamically regulates this microenvironment through high phenotypic plasticity (10). They typically polarize into either pro-inflammatory M1-like or anti-inflammatory/repair-associated M2-like subtypes. M1-like microglia secrete inflammatory mediators (e.g., TNF, IL-1β) and reactive oxygen species, exacerbating neurotoxicity. Conversely, M2-like microglia release anti-inflammatory cytokines (e.g., IL-10, TGF-β) to promote debris clearance, angiogenesis, and tissue remodeling (11, 12). Because the early and persistent dominance of M1-like polarization drives chronic inflammation and hinders regeneration, reversing this unfavorable immune state to restore a pro-regenerative equilibrium represents a promising clinical intervention strategy (13, 14).

In recent years, neuromodulation technologies have paved the way for new therapeutic approaches to SCI intervention (15, 16). One of the technologies that has piqued researchers’ interest is transcranial alternating current stimulation (tACS), known for being safe and non-invasive. Its unique advantage lies in its ability to modulate neural oscillations within specific frequency bands (17–19). By applying weak, frequency-specific alternating currents, tACS can effectively modulate cortical neural synchronization and network excitability. By leveraging intact or partially preserved corticospinal pathways, tACS can profoundly influence descending motor control functions and central neural plasticity (5). Beyond directly modulating neuronal activity, neuromodulation technologies have been shown to indirectly influence glial activation and inflammatory responses by regulating local metabolism, promoting neurotransmitter release, and mediating neuro-immune coupling (20, 21). However, in the context of SCI pathology, it remains unclear whether tACS can effectively remodel the polarization state of microglia, particularly regarding its regulatory role in the M1-like proinflammatory phenotype and its potential molecular mechanisms, and this warrants further investigation.

The signaling pathway involving cyclic guanosine monophosphate and protein kinase G is a key secondary messenger in the central nervous system. It plays a significant role in controlling essential physiological and pathological processes, including neuronal and glial excitability, local blood flow, cell survival, and the transcription of genes related to inflammation (22–24). The core molecule in this pathway, cGMP, is primarily synthesised by soluble guanylate cyclase (sGC) and is dynamically regulated by hydrolysis mediated by the phosphodiesterase (PDE) family. The downstream effector kinase PKG finely tunes cellular functions and signal integration by phosphorylating multiple substrate proteins (25, 26). The cGMP–PKG pathway is notably essential for both neural activity and immune cell activation, as well as for the release of inflammatory factors and oxidative stress responses. This implies that it could serve as an essential molecular mediator connecting electrical stimulation and neural activity to subsequent immune phenotypes. In terms of SCI specifically, this pathway may mediate the effects of interventions such as tACS on the polarization states of microglia. However, there have been few systematic studies to clarify whether the cGMP–PKG pathway directly participates in and mediates the regulatory role of tACS in converting the M1/M2 phenotype in post-SCI microglia, and its specific mechanisms require further elucidation.

In this study, we confirmed that tACS suppresses M1 polarization of microglia, alleviates neuroinflammation, and promotes neuronal survival, thereby improving motor function recovery post-SCI. Involvement of the cGMP-PKG signaling pathway is how the substance under investigation operates, thereby driving microglia to switch from a pro-inflammatory to an anti-inflammatory phenotype, and exerting neuroprotective and anti-inflammatory effects.

## 2 Methods

### 2.1 Proteomics sequencing

Peptide mixtures were analyzed using a label-free quantitative strategy via nano-liquid chromatography-tandem mass spectrometry (nanoLC-MS/MS). The raw data were processed using Proteome Discoverer 2.4.1.15. Protein identification was filtered with a false discovery rate (FDR) of 1% at both the peptide and protein levels.

A total of eight biological replicates (n = 4 per group) were analyzed. A total of 5,967 proteins were identified, of which 4,803 were successfully quantified. A Student’s t-test was performed to identify differentially expressed proteins (DEPs), setting a threshold of fold change >1.5 and p-value <0.05. This analysis yielded 343 DEPs (241 up-regulated and 102 down-regulated in the TACS group versus the SCI group). Functional enrichment analysis of these DEPs revealed that the ‘cGMP-PKG signaling pathway’ was a key target.

### 2.2 Cell culture

BV2 microglia and VSC4.1 cells were both procured from Procell (Wuhan, China). BV2 cells were cultured in dedicated medium, and high-glucose DMEM with 10% fetal bovine serum was used to maintain VSC4.1 cells. The incubation of both cell lines occurred at 37 °C with 5% CO_2_, with fresh medium replaced every 1–2 days to sustain cellular viability.

### 2.3 Co-culture of cells

For the establishment of a conditioned co-culture system with BV2 microglia and VSC4.1 cells, the activation of BV2 microglia was initiated by stimulating them with LPS (1 μg/mL) for 12 hours. The medium was then replaced with fresh LPS-free medium for a further 24 hours of culture, and the conditioned medium (CM) from this stage was collected. This conditioned medium was transferred to VSC4.1 cells, replacing their original medium for co-culture. After 24 hours, the survival and injury status of the VSC4.1 cells were assessed. To ensure that the observed effects on VSC4.1 cells were mediated by soluble factors released from BV2 microglia and not by the direct action of residual 8-Br-cGMP-AM, the BV2 cells were washed twice with fresh, LPS-free medium before the collection of CM.

### 2.4 Live/dead staining

VSC4.1 cells underwent three PBS washes following co-culture before being stained with the AM/PI dual-stain kit. Samples were incubated in the dark at room temperature for 20 minutes to differentiate between live and dead cells. After staining, using an inverted fluorescence microscope from ZEISS, Germany, fluorescence images were captured after cells were washed three times with PBS to remove any unbound dye.

### 2.5 Animal experiments

The research followed the National Institutes of Health Guide for the Care and Use of Laboratory Animals and received approval from the Experimental Animal Ethics Committee at the First Affiliated Hospital of Nanchang University. (Approval No.: CDYFY-IACUC202603GR008). Female Sprague-Dawley rats, aged 8 weeks and weighing 240–280 grams, were utilized in the experiments and were provided by Zhejiang Weitong Lifu Co., Ltd. in Zhejiang, China. The housing conditions for the animals included a 12-hour light-dark cycle, 25 °C temperature, and 50% relative humidity. Drinking water was treated with high-pressure sterilization, and a period of at least 7 days was provided for animals to acclimate to the environment prior to experimentation. SCI models were established according to established protocols: Anesthesia was induced in the rats with an intraperitoneal injection of 1% sodium pentobarbital at a dosage of 40 mg/kg, underwent T10 laminectomy to expose the spinal cord, and received 10 seconds of clamping with an aneurysm clamp to induce injury. The sham surgery group underwent spinal cord exposure without clamping. Postoperatively, SCI rats underwent twice-daily manual bladder emptying until spontaneous micturition resumed. The study involved randomly assigning 60 eligible rats to two experimental designs: (1) Baseline intervention group (n = 36): sham-operated group, SCI group, SCI + tACS group, SCI + Sham-tACS group;(2) Pharmacological Mechanism Group (n = 24): sham-operated group, SCI group, SCI + Sham-tACS group, SCI + tACS group, and SCI + tACS + KT5823 group. In the group that underwent SCI, tACS, and KT5823, daily intraperitoneal injections of the PKG inhibitor KT5823 (3 mg/kg, dissolved in 5% DMSO) commenced following tACS intervention and continued for 14 consecutive days; the remaining groups were given the same volume of a 5% DMSO solution as a control.

### 2.6 Transcranial alternating current stimulation

This study used tACS equipment (purchased from Jiangxi Brain Regulation Technology Development Co., Ltd.) to treat rats with spinal cord injuries under 2.5% isoflurane anesthesia. The stimulation frequency of 50 Hz, current intensity of 1 mA, stimulation duration of 20 minutes per session, once daily for 14 consecutive days; the anode was placed on the rat’s frontal lobe and the cathode on the lower thoracoabdominal region; circular non-woven fabric electrodes with a diameter of 1 cm were uses. The real stimulation group received a 1 mA sinusoidal alternating current for 20 minutes per session, once daily for 14 consecutive days, with the current decreasing linearly to 0 mA during the final 15 seconds of each session. These parameters were based on prior research. An intensity of 1 mA effectively modulates cortical excitability with a good safety profile, and 20 minutes of stimulation induces neuroplastic changes while minimizing the risk of adverse reactions. In the sham stimulation group, the current was increased to 1 mA during the initial 15 seconds and then reduced to 20 μA in the following 15 seconds as part of a simulated protocol. Over the course of 20 minutes, the minimal current was maintained before it slowly declined to 0 μA in the last 15 seconds. This sequence mimicked the initial sensation of real stimulation while avoiding significant neuromodulator effects.

### 2.7 Behavioral assessment

The assessment of motor function after SCI was conducted systematically using gait analysis and the Basso, Beattie, and Bresnahan (BBB) scale. Primarily indicating balance, coordination, and weight support during rat movement, the BBB score ranges from 0, meaning full paralysis, to 21, meaning normal motor function (27, 28). Evaluations took place on days 1, 3, 7, 14, 21 and 28 after injury to cover the critical time window from the acute phase to recovery, and to dynamically monitor the impact of tACS on functional recovery. On the 28th day after the injury, a footprint analysis was performed, which is a transitional phase between the subacute and chronic stages, when improvement in motor function stabilizes. This timing allows the role of tACS in long-term neural recovery to be assessed.

### 2.8 Hematoxylin and eosin staining

Spinal cord sections embedded in paraffin were deparaffinized using xylene twice for 3–5 minutes each, then rehydrated sequentially through ethanol solutions with concentrations of 100%, 95%, 80%, and 70%. Sections were subjected to an 8-minute hematoxylin stain after being rinsed with tap water. The slides were rinsed with tap water for 1–3 minutes following staining, stained with eosin for 1 minute, and then gently rinsed with tap water for 30 seconds. The sections were dehydrated in a graded ethanol series (70%, 80%, and 95% for 1 minute each; 100% ethanol twice for 2–3 minutes each), cleared in xylene (twice for 3–5 minutes each), mounted with neutral resin, and examined under an optical microscope (Nikon Eclipse E100, Nikon). Mounting was performed carefully to avoid bubble formation, and the slides were dried at room temperature prior to examination.

### 2.9 Nissl staining

Spinal cord tissue sections were sequentially dehydrated with 70%, 80%, 90%, and 100% ethanol, followed by xylene clearing to enhance staining affinity. Subsequently, the sections were immersed in Nissl stain for approximately 15 minutes, rinsed with distilled water to remove excess dye and improve clarity. Post-staining, the sections experienced further dehydration through graded ethanol, xylene clearing, and were ultimately sealed with a mounting medium. Using a fluorescence microscope, observations and analyses were performed (Nikon Eclipse C1).

### 2.10 Elisa

cGMP concentration was determined using the Jingmei (China) ELISA kit. All reagents (including capture antibody, detection antibody, enzyme-labeled secondary antibody, and substrate) were prepared strictly according to the manufacturer’s instructions. Test samples and standards were added to corresponding wells of the ELISA plate to establish a standard curve and ensure quantitative accuracy. Absorbance (OD values) was measured at 450 nm using a Labsystems Multiskan MS microplate reader after the reaction was complete. The cGMP concentrations in the samples were derived from the standard curve, and ImageJ software was utilized for data analysis.

### 2.11 Western blotting

After collecting cell and spinal cord tissue samples, RIPA lysis buffer facilitated the extraction of total protein. Following centrifugation of the lysate at 11,000× g for 20 minutes at 4 °C, the supernatant was obtained. Concentration was determined using a BCA protein quantification kit and the samples were adjusted to an equal volume for loading. The samples of protein were combined with loading buffer, denatured by heating to 100 °C for ten minutes, and then loaded onto SDS-polyacrylamide gels for electrophoretic separation. After a ten-minute denaturation at 100 °C, proteins were moved onto nitrocellulose (NC) membranes. The membranes were blocked with 5% skimmed milk powder at room temperature for an hour to minimize non-specific binding. Overnight incubation of the primary antibody was carried out at 4 °C using the listed antibodies and their respective dilutions: PKG (Immunoway, 1:850); iNOS (Abcam, 1:850); Iba-1 (CST, 1:850); IL-6 (Affinity, 1:850); IL-1β (Proteintech, 1:850); CD11b (Abcam, 1:850); COX-2 (Abmart, 1:850); Arg-1 (Immunoway, 1:850); BAX (Proteintech, 1:850); Bcl-xL (CST, 1:850); GAP43 (CST, 1:850); SYP (CST, 1:850); PSD95 (CST, 1:850). After TBST washing on day two, the membrane was incubated at room temperature for 75 minutes with a horseradish peroxidase (HRP)-labeled secondary antibody (ZSGB-BIO) diluted 1:10,000. Following chemiluminescent substrate development, band intensities were quantified using ImageJ software.

### 2.12 Immunofluorescence staining

The fixation of tissue sections and cells with paraformaldehyde occurred before antigen retrieval. This was followed by blocking with 5% bovine serum albumin (BSA) for 25–35 minutes to block non-specific binding sites and diminish background signal. Following this, the primary antibody mixture was applied: CD11b, Abcam, 1:450; Bcl-xL, CST, 1:450; Arg-1, Proteintech, 1:450; Iba-1, CST, 1:350; IL-1β, Abcam, 1:350) was applied and left overnight at 4 °C in a moisture-rich environment shielded from light. The following day, incubation continued for one hour at room temperature in the dark to enhance antibody binding. To enhance cellular morphology visualization, co-staining with Phalloidin (labelling F-actin) and Bcl-xL was employed. Subsequently, DAPI nuclear stain was added dropwise and incubated for 10 minutes at room temperature in the dark to label cell nuclei. Following staining completion, a self-fluorescence quenching reagent was applied for 5 minutes to suppress background fluorescence. Finally, using a fluorescence microscope, images were taken after sealing the slides with an anti-fade mounting medium (Nikon Eclipse C1).

### 2.13 q-PCR

An RNA extraction kit was used to extract total RNA from BV2 cells. Using SYBR Green reagent, qPCR was carried out after synthesizing cDNA via reverse transcription. Amplification reactions were conducted on a CFX Connect real-time qPCR instrument (Bio-Rad), with procedures strictly adhering to the manufacturer’s protocol. The internal control was GAPDH, and the 2^−ΔΔCT method was employed for relative quantification. See **Supplementary Table S1** for primer sequences.

### 2.14 Statistical analysis

A one-way or two-way ANOVA was first performed to determine if there were notable differences between the groups, and this was followed by a Tukey *post hoc* test. To identify significant differences among groups when the data were not normally distributed, the Kruskal-Walli’s test was applied. The data were represented as mean ± standard deviation (SD), and a p-value below 0.05 was considered statistically significant.

## 3 Results

### 3.1 cGMP-PKG pathway alleviates neuroinflammation by inhibiting M1 microglial polarization

In recent years, tACS has demonstrated significant potential as a non-invasive neuromodulation technique for SCI. In this study, tACS was administered to rats with SCI, with the anode positioned on the frontal-parietal region and the cathode on the ventral thoracic region(**Figure 1A**). The rats received 20 minutes of stimulation daily for 14 consecutive days. To elucidate the therapeutic effects of tACS on SCI and its potential molecular mechanisms systematically, a quantitative proteomics analysis was performed on spinal cord tissue from the tACS intervention group and the control group which received sham stimulation. Pathway enrichment analysis (including string diagram clustering and scatter plot visualization) revealed that differentially expressed proteins were significantly enriched in ‘pathways of neurodegeneration’ and the ‘cGMP-PKG signaling pathway’(**Figures 1B, C**). Furthermore, by integrating the results from Venn diagrams, volcano plots, and quantitative analysis, we identified 343 differentially expressed proteins (DEPs) in the tACS intervention group compared to the SCI group. Further analysis revealed that 123 of these proteins exhibited statistical significance, with 77 significantly up-regulated and 46 significantly down-regulated (**Figures 1D, E**). Taken together, these findings suggest that tACS may mediate neurogenic effects by activating the cGMP-PKG signaling pathway, thereby promoting synaptic functional remodeling and neural repair. However, the precise molecular mechanisms by which tACS may regulate the cGMP-PKG pathway and influences neurogenesis remain to be elucidated.

**Figure 1:**
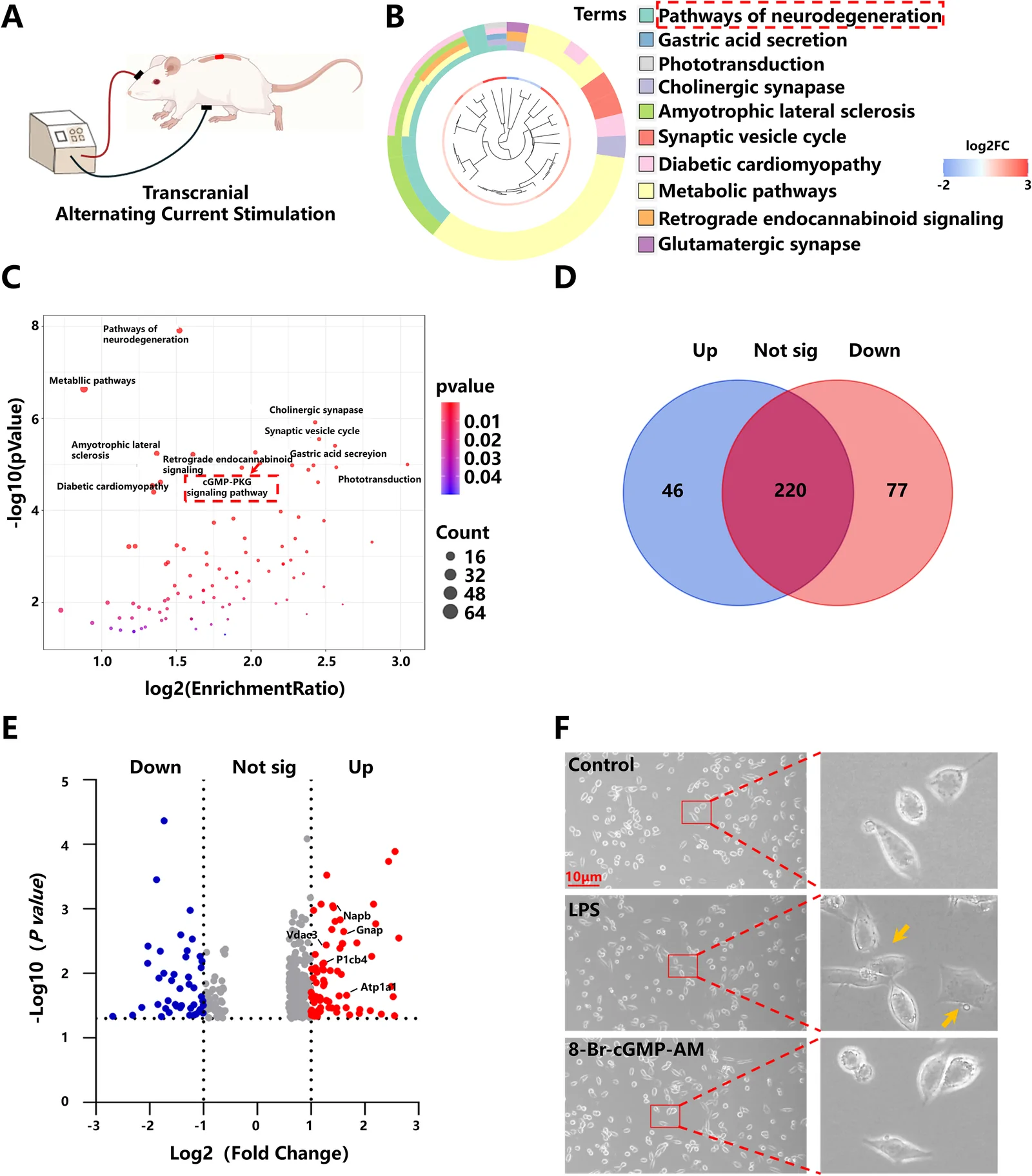
Proteomic findings and microglial morphological remodeling following tACS. **(A)** Schematic diagram showing tACS treatment in rats with SCI. **(B, C)** Pathway enrichment analysis following proteomics sequencing, visualized via chord and scatter plots, revealing significantly enriched biological pathways and their interrelationships. **(D, E)** Statistical analysis of proteomics sequencing data was used to generate venn diagram and volcano plots, providing a visual representation of significant differences in protein expression levels between different groups. **(F)** Morphological observations of BV2 microglia in each group. Yellow arrows indicate amoeboid-like morphology in microglia following LPS stimulation, reflecting cellular characteristics under inflammatory conditions (scale bar = 10 μm). After verifying that the data followed a normal distribution, a one-way or two-way analysis of variance (ANOVA) was used to assess differences between groups, supplemented by Tukey’s *post-hoc* test. Data are displayed as mean ± SD, with each experiment independently performed at least three times. *p < 0.05, **p < 0.01.

Previous studies have demonstrated that non-invasive neuromodulation techniques can alleviate neuroinflammatory responses by enhancing the function of the brain’s neural networks, diminishing the output of pro-inflammatory cytokines, and modulating immune cell activity (29–31). Furthermore, the cGMP-PKG pathway exerts significant negative regulation during inflammation, and its involvement aids inflammatory resolution and preserves tissue function (32–34). Against this backdrop, we established an M1 BV2 microglial inflammatory model *in vitro* using lipopolysaccharide (LPS), employing the PKG activator 8-Br-cGMP-AM as an intervention to investigate whether the cGMP-PKG pathway could inhibit microglial M1 polarization and exert anti-inflammatory effects. Experiments revealed that BV2 cells exhibited amoeboid morphological changes 24 hours after LPS stimulation, a hallmark of M1 polarization. In contrast, cells pretreated with 8-Br-cGMP-AM for 24 hours prior to LPS stimulation showed no significant morphological alterations (**Figure 1F**; **Supplementary Figure S1A**). Further Western blot analysis demonstrated that 8-Br-cGMP-AM treatment significantly reduced the levels of protein expression of iNOS, COX-2 and IL-1βinduced by LPS in M1-polarised microglia (**Figures 2A–E**).qPCR results indicated that 8-Br-cGMP-AM significantly downregulates the mRNA expression of LPS-induced M1 markers (including iNOS, COX-2, TNF and IL-1β) compared to the LPS group (**Figures 2F–I**). Immunofluorescence staining for CD11b and Iba-1 also supported this conclusion, showing that involvement of the cGMP-PKG pathway has a significant anti-inflammatory effect (**Figures 2J–L**). Taken together, these findings reveal the crucial role of the cGMP-PKG pathway in suppressing microglial M1 polarization and highlight its potential therapeutic value in combating neuroinflammation.

**Figure 2:**
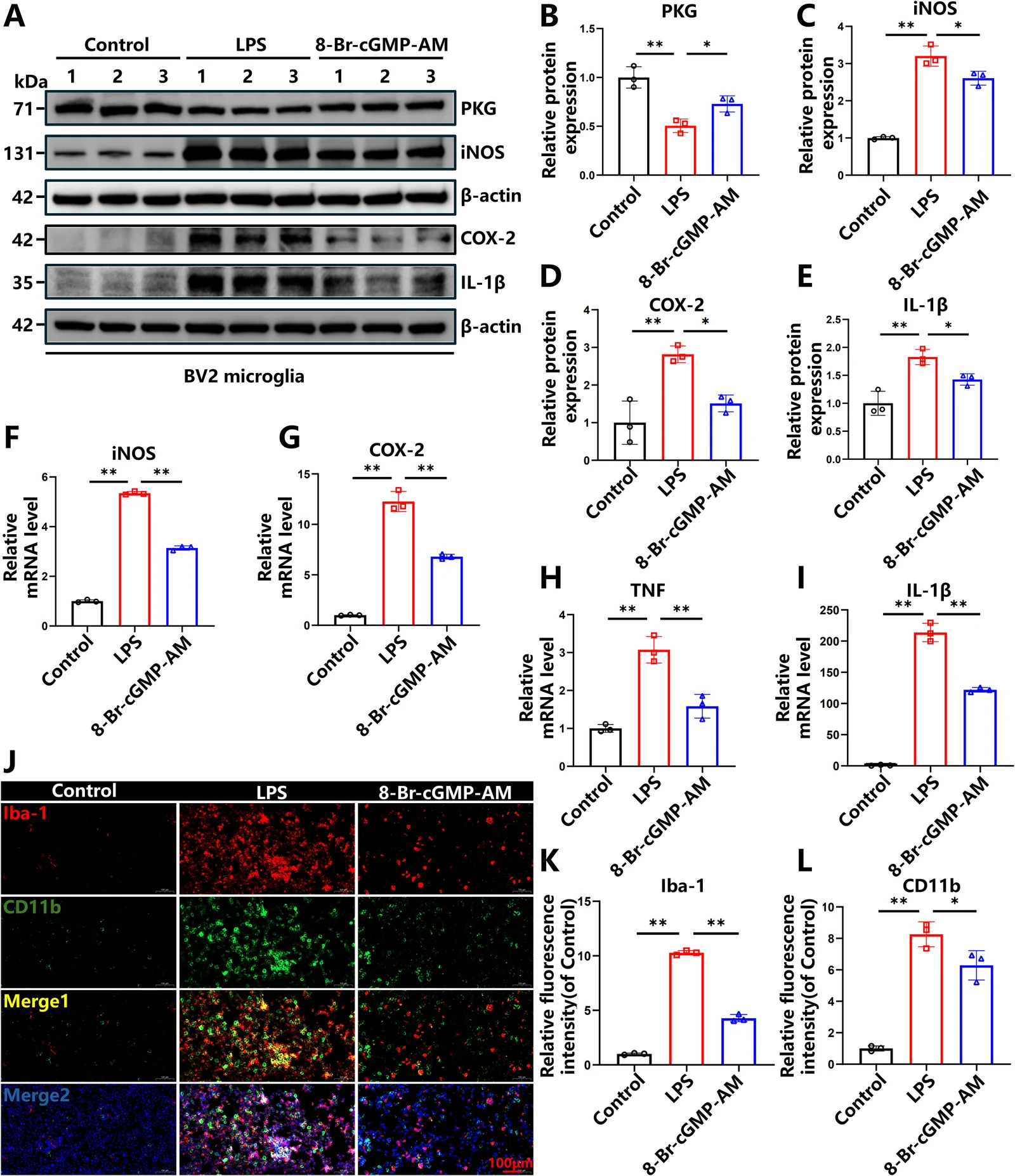
cGMP-PKG pathway alleviates neuroinflammation by inhibiting M1 microglial polarization. **(A–E)** Western blot detection and quantitative analysis revealed changes in PKG protein levels and in the expression of M1-type microglia markers (iNOS, COX-2 and IL-1β). **(F–I)** qRT-PCR detected the mRNA expression levels of the M1-associated inflammatory factors iNOS, COX-2, TNF and IL-1β. **(J–L)** Double immunofluorescence staining and quantitative analysis of iNOS and Iba-1 expression reflecting the activation status and polarization characteristics of microglia (scale bar = 100 μm). After verifying that the data followed a normal distribution, a one-way or two-way analysis of variance (ANOVA) was used to assess differences between groups, supplemented by Tukey’s *post-hoc* test. Data are displayed as mean ± SD, with each experiment independently performed at least three times. *p < 0.05, **p < 0.01.

### 3.2 cGMP-PKG pathway inhibits microglia M1 polarization mediated neuronal death

Studies in the past have indicated that M1 microglia have neurotoxic effects by emitting significant levels of pro-inflammatory cytokines, including TNF and IL-1β, thus intensifying neuronal harm (35, 36). According to these findings, we theorize that activating the cGMP-PKG pathway could reduce neurotoxicity caused by M1-polarised microglia by preventing their phenotypic shift towards the M1 state and decreasing neuroinflammation. To validate this hypothesis, we established an *in vitro* conditioned medium (CM) co-culture system. First, BV2 microglia were stimulated with LPS to induce M1 polarization. The intervention group was pre-treated with the PKG activator 8-Br-cGMP-AM. Subsequently, conditioned medium from each group was collected for culturing VSC4.1 spinal motor neurons (**Figure 3A**). Western blot analysis revealed that the conditioned medium secreted by the 8-Br-cGMP-AM pre-treated microglia significantly increased the expression of the anti-apoptotic protein Bcl-xL in the VSC4.1 neurons (**Figures 3B, C**; *p*<0.05). Immunofluorescence staining verified this discovery, which revealed enhanced Bcl-xL fluorescence signals in the 8-Br-cGMP-AM intervention group(**Figures 3D, E**; *p*<0.01). Furthermore, live/dead cell staining (Calcein-AM/PI) showed that 8-Br-cGMP-AM pre-treatment significantly reversed the cytotoxic effects of LPS-activated microglia on VSC4.1 neurons, thereby improving neuronal survival rates(**Figures 3F, G**; *p*<0.05). In summary, these outcomes suggest that by activating the cGMP-PKG pathway, neuronal injury can be effectively reduced through the inhibition of M1 microglia polarization and the neuroinflammation they mediate, offering significant neuroprotection.

**Figure 3:**
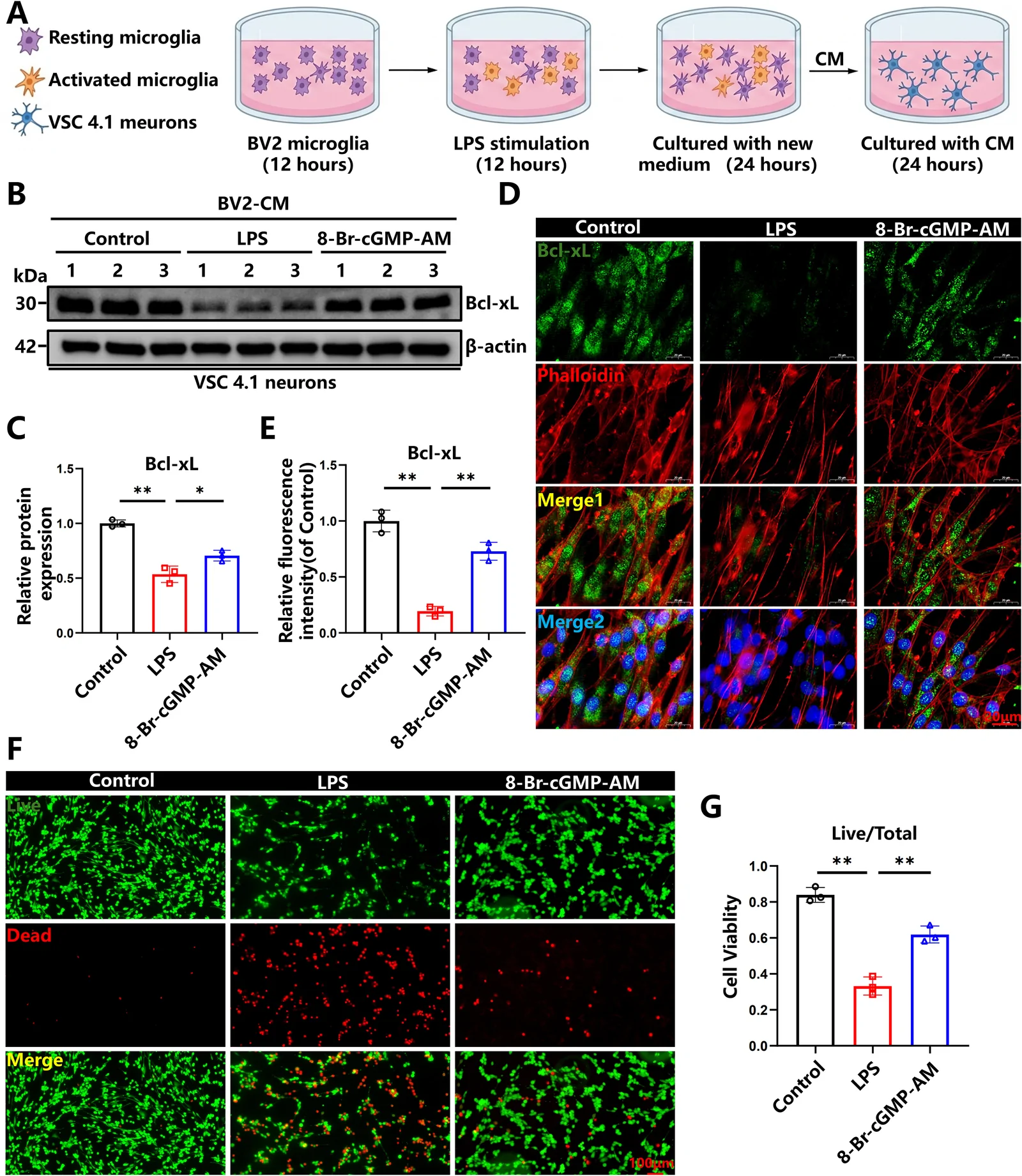
cGMP-PKG pathway inhibits microglia M1 polarization mediated neuronal death. **(A)** Experimental schematic: VSC4.1 neuronal cells were cultured in a conditioned medium derived from microglia that had been treated under different conditions, to assess the impact of this on neuronal survival. **(B, C)** Western blot analysis for detecting and quantifying Bcl-xL protein expression related to anti-apoptosis. **(D, E)** Bcl-xL immunofluorescence staining (green) combined with phalloidin (red) labelling of the cytoskeleton and cell outline for colocalisation observation and fluorescence intensity quantification (scale bar = 20 μm). **(F, G)** Live/dead cell dual staining (Calcein-AM/PI) was used to evaluate the survival rate of VSC4.1 neurons, followed by statistical quantification (scale bar = 100 μm). After verifying that the data followed a normal distribution, a one-way or two-way analysis of variance (ANOVA) was used to assess differences between groups, supplemented by Tukey’s *post-hoc* test. Data are displayed as mean ± SD, with each experiment independently performed at least three times. *p < 0.05, **p < 0.01.

### 3.3 tACS promotes recovery of hind limb motor function in rats with SCI

To evaluate the effect of tACS on motor recovery in rats with SCI, we conducted Basso, Beattie and Bresnahan (BBB) motor assessments on postoperative days 0, 1, 3, 7, 14, 21 and 28, supplemented by footprint analysis on day 28 (**Figure 4A**). The results showed that, on both days 14 and 28, the tACS group exhibited significantly higher BBB scores than the SCI group and the sham tACS group (**Figures 4B–D**). Footprint analysis revealed that, by day 28, both the SCI and sham tACS groups showed hindlimb footprints that were significantly blurred and dragged. Although mild dragging persisted in the tACS group, there was significant improvement in footprint clarity and gait coordination (**Figure 4E**). Quantitative gait parameter analysis revealed that stride length and support base in the tACS group were significantly shorter than in the SCI group and sham tACS group (**Figures 4F, G**). Overall, tACS intervention significantly promoted motor recovery in SCI rats.

**Figure 4:**
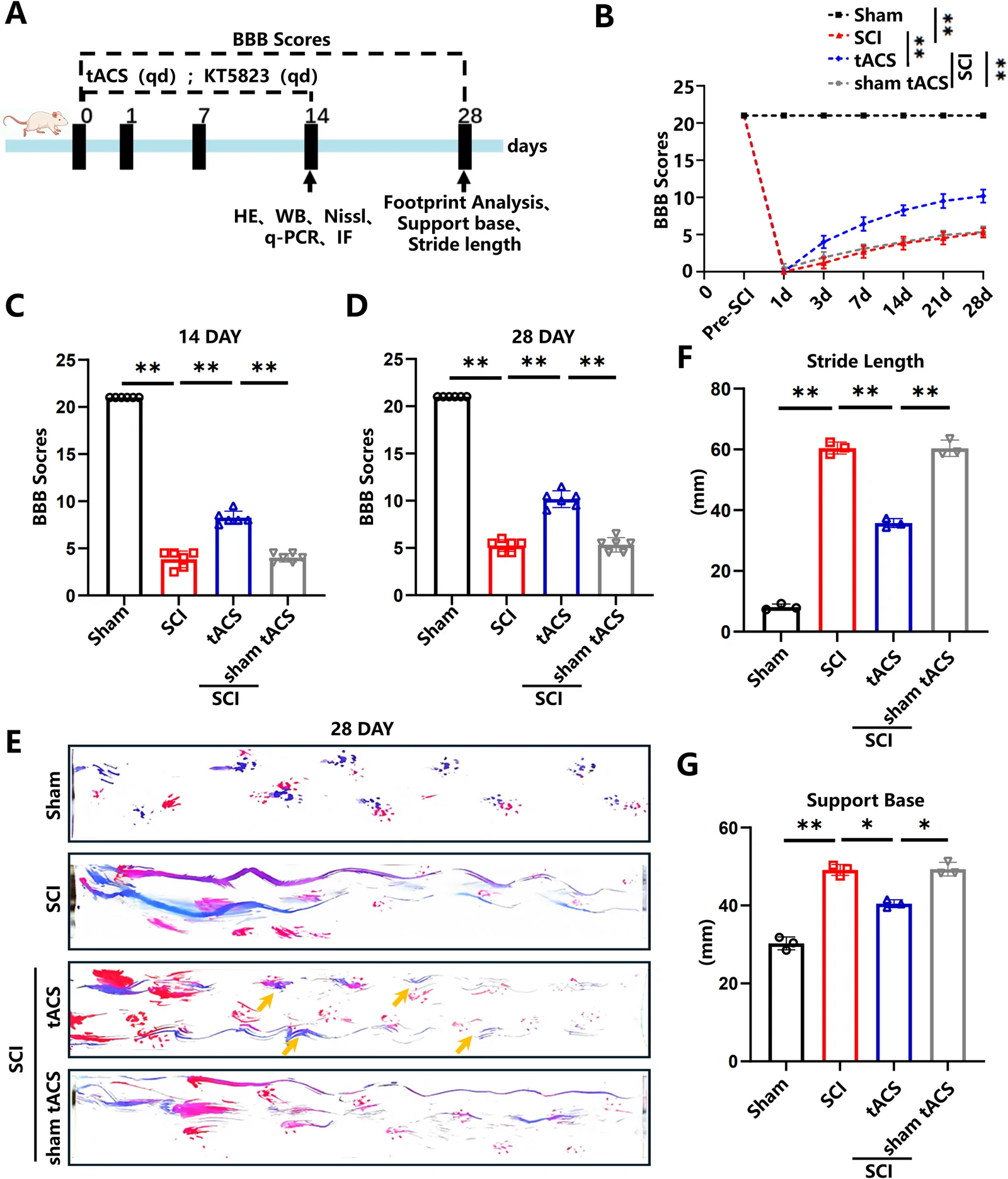
tACS promotes recovery of hind limb motor function in rats with SCI. **(A)** Schematic timeline of the *in vivo* animal experiments. **(B)** Dynamic changes in BBB scores reflecting recovery of hindlimb motor function in each rat group over 28 days post-surgery. **(C, D)** Statistical results of BBB scores on days 14 and 28 after surgery for the sham group, the SCI group and the SCI + tACS group. **(E)** Footprint analysis of animals in all three groups at 28 days post-surgery. Red footprints represent the forelimbs, and blue footprints represent the hindlimbs. Yellow arrows indicate where the first coordinated hindlimb footprints appeared in the tACS group. **(F, G)** Quantitative assessment of motor function recovery in each group at 28 days post-SCI, based on stride length and support base. After verifying that the data followed a normal distribution, a one-way or two-way analysis of variance (ANOVA) was used to assess differences between groups, supplemented by Tukey’s *post-hoc* test. Data are displayed as mean ± SD, with each experiment independently performed at least three times. *p < 0.05, **p < 0.01.

### 3.4 tACS inhibited microglia M1 polarization and reduced inflammation after SCI

To systematically assess the impact of tACS on microglial activation after SCI, spinal cord tissue was collected from the lesion site of rats in each group on the 14th day after surgery for Western blot analysis. The findings indicated that, in comparison to the SCI and sham tACS groups, the tACS group exhibited significantly downregulated expression of the M1 microglia markers iNOS, Iba-1, COX-2 and IL-6 (**Figures 5A–E**), while the M2 marker Arg-1 was significantly upregulated (**Figures 5F, G**). These outcomes imply that tACS is effective in decreasing M1 polarization of microglia and supports a move towards an anti-inflammatory phenotype, which helps in easing neuroinflammation. Considering the essential function of the cGMP-PKG signaling pathway in controlling neuronal survival, plasticity, and neuroprotection, we explored if this pathway is involved in the anti-inflammatory effects of tACS. The findings indicated a notable decrease in cGMP and PKG expression in the SCI group and the sham tACS group. Notably, after 14 days of tACS intervention, cGMP and PKG protein levels significantly recovered (**Figures 5H, J**). In conclusion, tACS may exert anti-neuroinflammatory effects by activating the cGMP-PKG signaling pathway and inhibiting M1 polarization of microglia.

**Figure 5:**
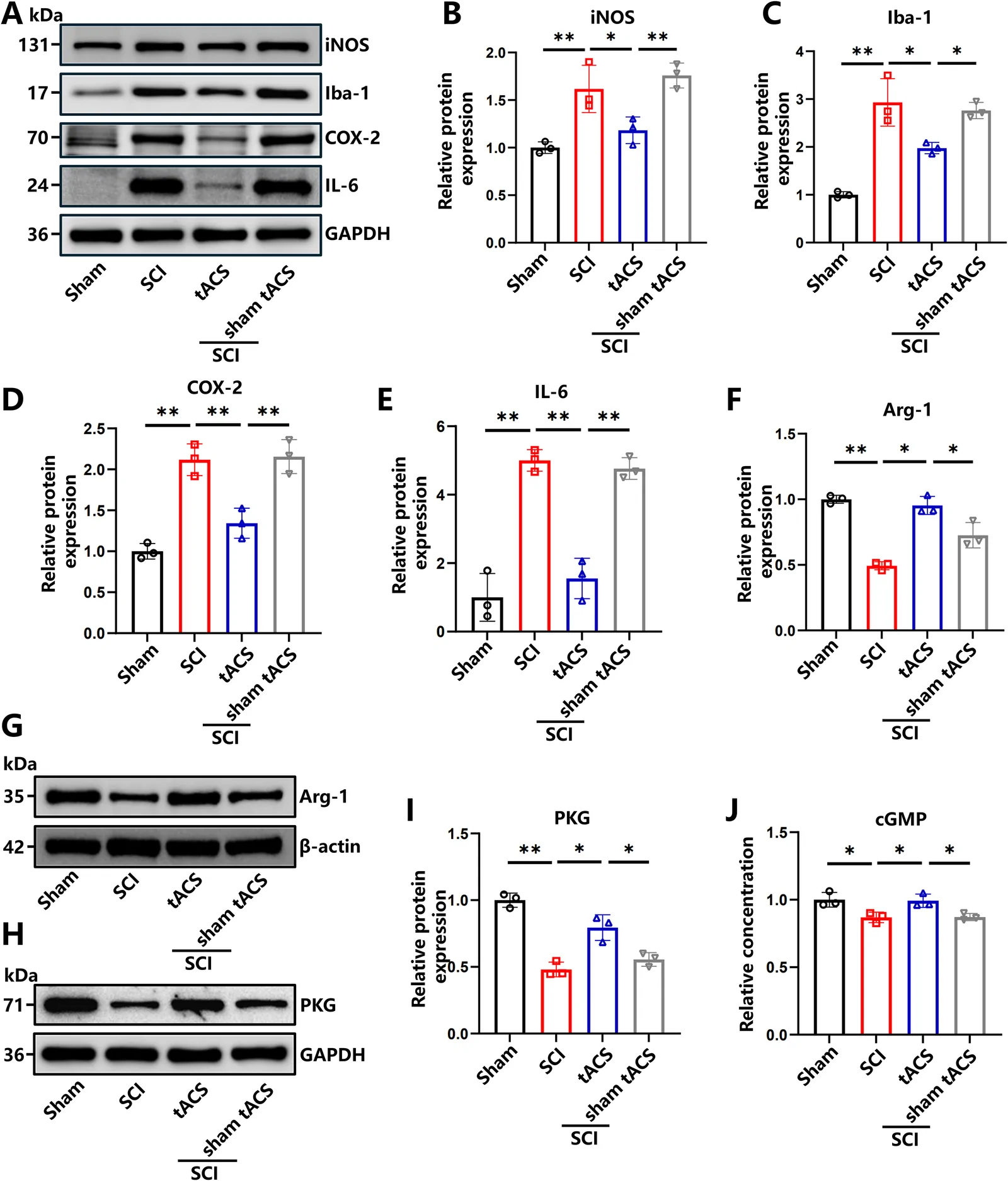
tACS inhibited microglia M1 polarization and reduced inflammation after SCI. **(A–E)** Western blot analysis of protein expression levels for M1 microglia markers (iNOS, COX-2 and IL-6) and the universal microglia activation marker Iba-1, with quantitative assessment. **(F, G)** Western blot analysis of Arg-1 protein expression levels as an M2 microglia marker, alongside the corresponding quantitative results. **(H–J)** ELISA and Western blot assays to evaluate cGMP–PKG signaling pathway involvement following tACS intervention, respectively measuring cGMP content and PKG protein expression. After verifying that the data followed a normal distribution, a one-way or two-way analysis of variance (ANOVA) was used to assess differences between groups, supplemented by Tukey’s *post-hoc* test. Data are displayed as mean ± SD, with each experiment independently performed at least three times. *p < 0.05, **p < 0.01.

### 3.5 tACS inhibited microglia M1 polarization and reduced inflammation after SCI via cGMP-PKG signaling pathway

To delve deeper into the involvement of the cGMP-PKG signaling pathway in the anti-inflammatory actions facilitated by tACS, the PKG-specific inhibitor KT5823 was administered via daily intraperitoneal injection to SCI rats for 14 consecutive days following tACS intervention, to block pathway activity. Western blot analysis revealed that KT5823 treatment significantly reduced PKG protein expression in spinal cord tissue (**Figures 6A, B**), confirming effective pathway inhibition. Alongside the downregulation of PKG activity, the expression of the M2-type microglia marker Arg-1 was also significantly decreased (**Figures 6C, D**). This finding was further validated by Iba-1/Arg-1 double immunofluorescence staining: following KT5823 intervention, Arg-1 co-localization fluorescence signals within Iba-1^+^ microglia markedly diminished, indicating the reversal of tACS-induced M2 polarization (**Figures 6E, F**). Furthermore, the M1 proinflammatory phenotype was markedly increased by the inhibition of the cGMP-PKG pathway. The analysis using Western blot revealed a marked increase in protein expression of iNOS, COX-2, IL-6 and the microglial activation marker Iba-1 (**Figures 7A–E**). Iba-1/IL-1β double-staining experiments further demonstrated that KT5823 treatment significantly increased IL-1β fluorescence intensity in activated microglia, confirming reactivation of neuroinflammatory responses (**Figure 7F**; **Supplementary Figure S2A**). In summary, these findings suggest that tACS inhibits M1 polarization of microglia via the cGMP-PKG signaling pathway, thereby exerting anti-neuroinflammatory effects. This further supports the potential value of tACS as a neuroprotective intervention.

**Figure 6:**
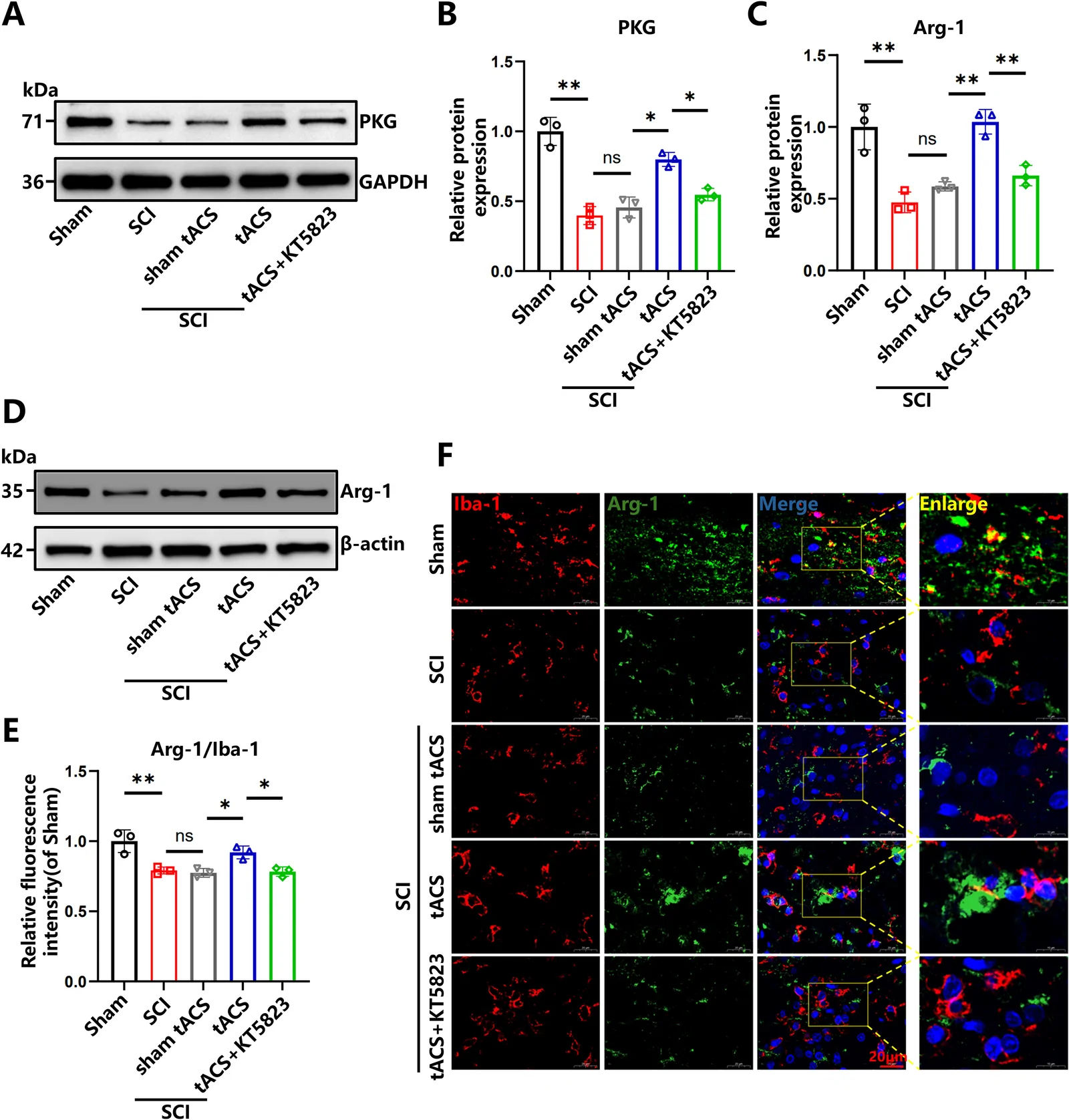
tACS promotes the M2 polarization of microglia via the cGMP-PKG signaling pathway. **(A, B)** Western blot detection and quantitative analysis revealed that PKG protein expression levels were significantly reduced following the administration of the PKG inhibitor KT5823 after tACS. **(C, D)** Western blot detection and quantitative analysis showed that the expression of the M2 microglia marker Arg-1 decreased significantly following tACS treatment combined with KT5823 administration. **(E, F)** Double immunofluorescence staining and quantitative analysis showed that injection of KT5823 after tACS significantly reduced the co-expression of Arg-1 and Iba-1, suggesting the inhibition of M2 microglia activation (scale bar = 20 μm). After verifying that the data followed a normal distribution, a one-way or two-way analysis of variance (ANOVA) was used to assess differences between groups, supplemented by Tukey’s *post-hoc* test. Data are displayed as mean ± SD, with each experiment independently performed at least three times. *p < 0.05, **p < 0.01.

**Figure 7:**
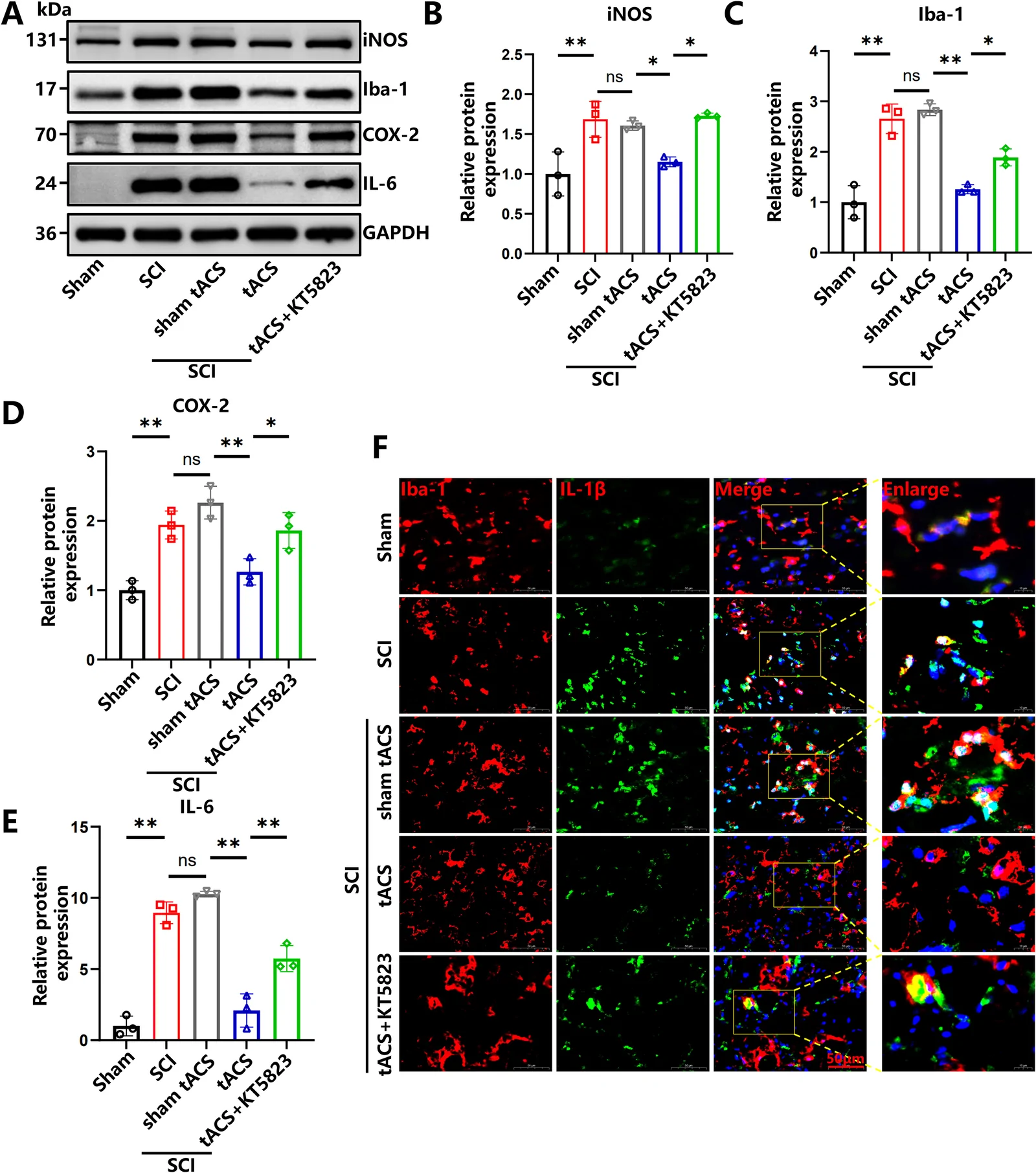
tACS inhibited microglia M1 polarization via cGMP-PKG signaling pathway. **(A–E)** Western blot detection and quantitative analysis revealed that, following tACS treatment, the administration of the PKG inhibitor KT5823 significantly increased the expression levels of the following M1-type microglial markers: iNOS, COX-2 and IL-6. It also increased the expression of the universal microglial activation marker Iba-1. **(F)** Double immunofluorescence staining showed that the IL-1β to Iba-1 fluorescence ratio increased significantly following combined tACS and KT5823 treatment, indicating enhanced M1 polarization (scale bar = 50 μm). After verifying that the data followed a normal distribution, a one-way or two-way analysis of variance (ANOVA) was used to assess differences between groups, supplemented by Tukey’s *post-hoc* test. Data are displayed as mean ± SD, with each experiment independently performed at least three times. *p < 0.05, **p < 0.01.

SCI causes localized tissue destruction, triggering cell death and inflammatory responses that further damage the structural integrity of the surrounding neural tissue (37). The initial injury often results in the formation of cavities within the spinal cord that progressively enlarge over time, exacerbating functional deficits (38, 39). To evaluate the therapeutic efficacy of tACS, we quantitatively analyzed the cavity area within the injured zone via HE staining on postoperative day 14. The results revealed significant cavitation in the SCI group as well as the sham tACS group. In contrast, tACS intervention markedly reduced cavity extent and mitigated tissue damage. Notably, co-treatment with the PKG inhibitor KT5823 significantly increased cavity area, suggesting that it reverses the protective effects of tACS (**Figures 8A, B**). As post-SCI elevation of reactive oxygen species (ROS) can cause neuronal injury and death, we also assessed neuronal morphology via Nissl staining. Significant cytoplasmic atrophy and nuclear condensation were observed in neurons from the SCI and sham tACS groups, whereas neurons in the tACS group retained relatively intact structures, indicating a protective effect on neurons. The worsening of these pathological changes by KT5823 treatment further confirms the important role of the cGMP-PKG pathway in repair facilitated by tACS (**Figures 8C, D**). To investigate whether neuronal death was related to apoptosis, our investigation focused on the expression of Bcl-xL, known for its anti-apoptotic role, and BAX, known for its pro-apoptotic role, in the injured zone. Western blot analysis revealed that tACS significantly increased Bcl-xL expression and decreased BAX expression, while KT5823 administration completely reversed these effects (**Figures 8E–G**). In summary, tACS effectively promotes spinal cord tissue repair and reduces neuronal apoptosis by activating the cGMP-PKG signaling pathway. The protective effect is hindered by PKG inhibition, which further supports the importance of this pathway in tACS’s neuroprotective mechanism.

**Figure 8:**
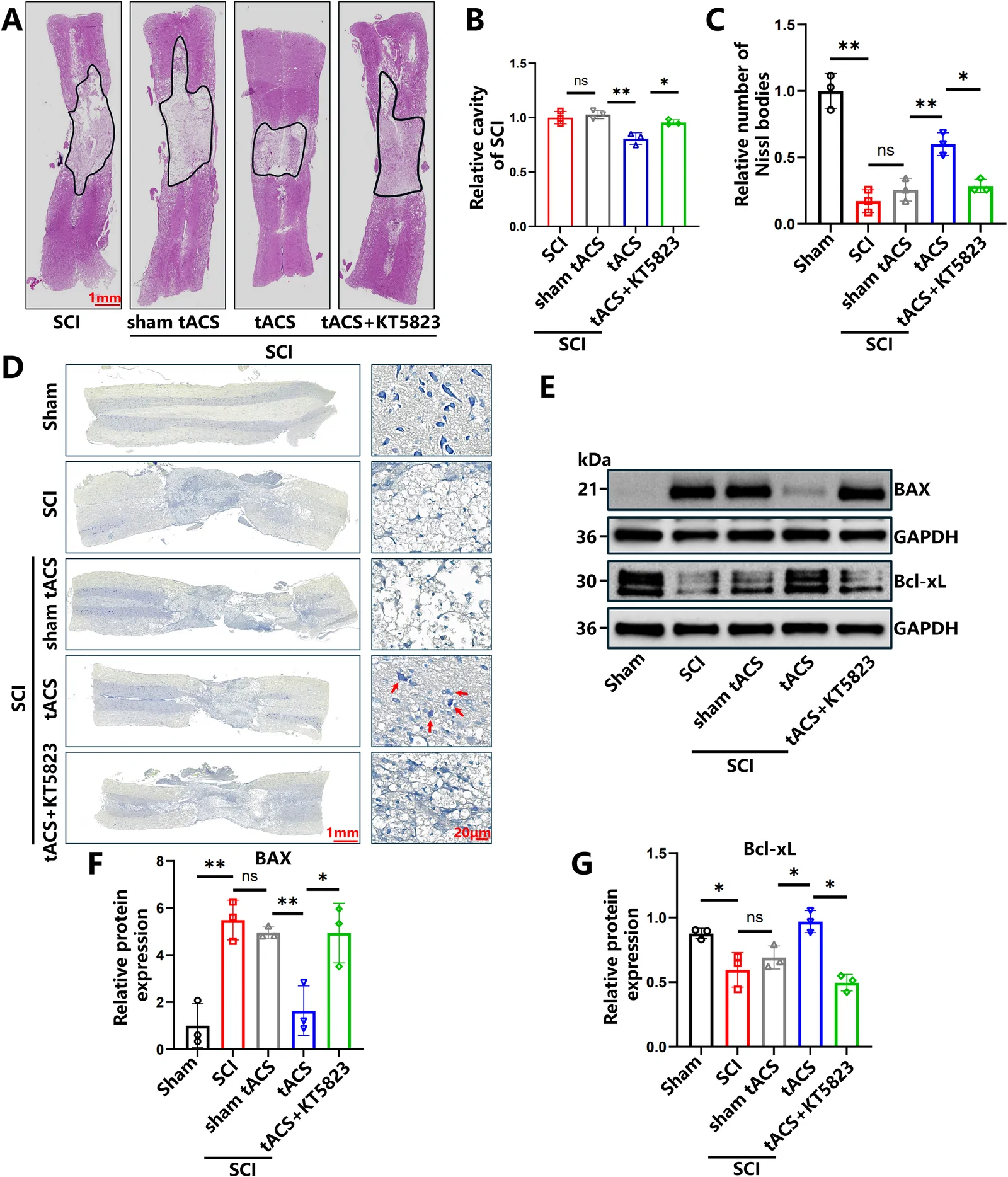
tACS promotes tissue repair and attenuates neuronal death via the cGMP-PKG signaling pathway. **(A, B)** HE was used to evaluate and analyze quantitatively the cavitation area at the injury site in each group following the administration of the PKG inhibitor KT5823 (scale bar = 1 mm). **(C, D)** Nissl staining was used to detect neuronal survival in each group after KT5823 administration, and quantitative analysis was used to reflect the extent of neuronal death (scale bar = 1 mm). **(E–G)** Western blotting was used to detect the expression levels of the pro-apoptotic protein Bax and the anti-apoptotic protein Bcl-xL. Quantitative analysis was then used to evaluate the activation status of neuronal death. After verifying that the data followed a normal distribution, a one-way or two-way analysis of variance (ANOVA) was used to assess differences between groups, supplemented by Tukey’s *post-hoc* test. Data are displayed as mean ± SD, with each experiment independently performed at least three times. *p < 0.05, **p < 0.01.

### 3.6 tACS promotes motor function recovery and synaptogenesis via the cGMP-PKG signaling pathway with good safety

Finally, we investigated the effect of tACS on the recovery of motor function using the BBB motor score and gait analysis and examined whether the cGMP-PKG pathway was involved. The results showed that the PKG inhibitor KT5823 significantly reduced the therapeutic effect of tACS. The tACS+KT5823 group had significantly reduced BBB scores on postoperative days 14 and 28 compared to the tACS group (**Figures 9A–C**). Further footprint analysis revealed that KT5823 treatment exacerbated hindlimb dragging and impaired gait coordination. Quantitative gait parameters showed significant increases in support base and stride length in the tACS+KT5823 group, suggesting a reversal of motor function (**Figures 9D–F**). As motor recovery critically depends on neural regeneration and synaptic reconstruction, we examined the expression of the neural regeneration marker GAP43 (40), as well as the synapse-associated proteins SYP (41) and PSD95 (42). We revealed that injury markedly reduced GAP43, SYP and PSD95 levels, with no significant improvement observed following sham tACS intervention. Conversely, tACS treatment significantly increased the expression of all three proteins. Notably, KT5823 completely reversed the upregulation of GAP43, SYP and PSD95 induced by tACS (**Figure 9G**; **Supplementary Figures S3A–C**). To assess the biosafety of tACS, major organs (heart, liver, spleen, lungs, and kidneys) were harvested on day 28 post-surgery for HE staining. The results revealed no significant pathological alterations in the structure of organ tissue between the tACS and SCI groups (**Figure 9H**), indicating favorable safety profiles for tACS under these experimental conditions. In summary, by activating the cGMP-PKG signaling pathway, tACS effectively boosts neuroregeneration and synapse development. This reduces neuronal damage and significantly improves the recovery of motor function in rats with SCI. This approach demonstrates promising therapeutic potential and safety, with no evident systemic toxicity.

**Figure 9:**
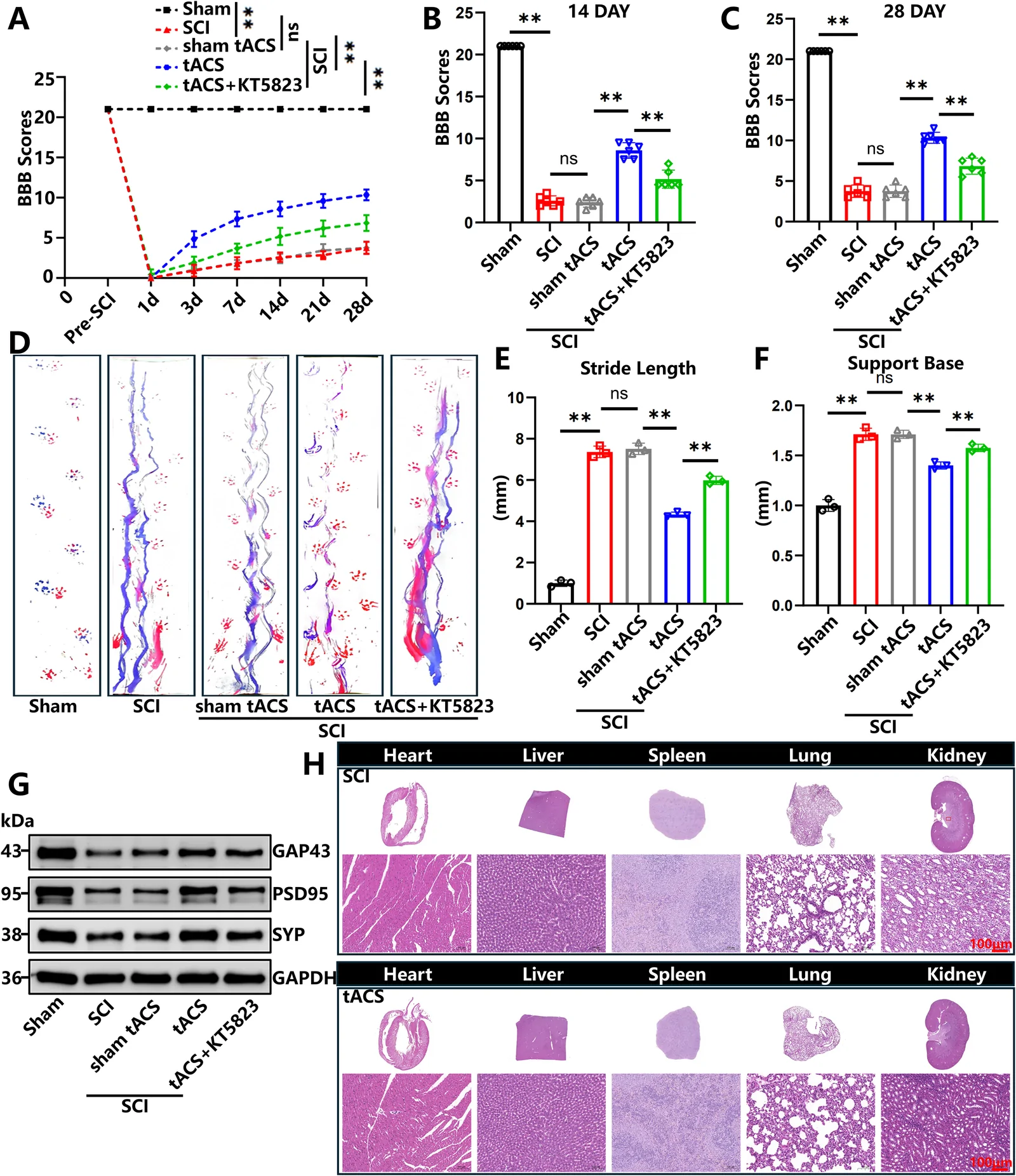
tACS promotes motor function recovery and synaptogenesis via the cGMP-PKG signaling pathway with good safety. **(A)** Dynamic curves showing the recovery of hindlimb motor function in rats across groups at postoperative days 1, 3, 7, 14, 21 and 28 following tACS combined with the PKG inhibitor KT5823. **(B, C)** Quantitative analysis of BBB scores on postoperative days 14 and 28 following the administration of combined tACS+ KT5823 group. **(D)** Footprint diagrams on postoperative day 28, where red indicates forelimb footprints and blue indicate hindlimb footprints, are used to assess gait coordination. **(E, F)** Quantitative analysis of stride length and support base width across groups to evaluate the role of the cGMP–PKG signaling pathway in tACS-mediated motor recovery. **(G)** Western blot detection of the expression levels of the synaptic-associated proteins GAP-43, PSD-95 and SYP to investigate whether tACS promotes neural synapse remodeling after SCI by activating the cGMP–PKG pathway. **(H)** HE-stained sections of major organs, including the heart, liver, spleen, lungs and kidneys, were evaluated to assess the impact of the tACS intervention on the morphology and structure of systemic organs (scale bar = 100 μm). After verifying that the data followed a normal distribution, a one-way or two-way analysis of variance (ANOVA) was used to assess differences between groups, supplemented by Tukey’s *post-hoc* test. Data are displayed as mean ± SD, with each experiment independently performed at least three times. *p < 0.05, **p < 0.01.

## 4 Discussion

This study systematically addressed the critical question of whether tACS can improve the repair of SCI by modulating the inflammatory phenotype of microglia. Following SCI, we demonstrated that tACS significantly inhibits the polarization of microglia towards an M1-like proinflammatory phenotype. It also reduces inflammatory cytokine levels and mitigates the deterioration of the neuroinflammatory microenvironment. Furthermore, it enhances neuronal survival and histological injury repair, ultimately promoting motor function recovery. Furthermore, the essential role of the cGMP-PKG signaling pathway in these effects was discovered and validated through a combination of proteomics enrichment analysis and pharmacological blockade experiments. Our data uncovers a sequential signaling axis wherein electrical stimulation triggers neural network and neuroimmune coupling, leading to cGMP-PKG activation, microglial phenotype conversion, and ultimately, neuroprotection and functional recovery.

### 4.1 tACS reprograms the neuroinflammatory microenvironment following SCI

Secondary injury following SCI is essentially a cascade of pathological processes centered on the abnormal amplification of immune-inflammatory responses (43, 44). Extensive research indicates that, in the early phase after injury, M1-like microglia dominate the inflammatory landscape persistently (45). These cells exacerbate neuronal apoptosis, disrupt the structural integrity of synapses, and inhibit axonal regeneration and functional remodeling by releasing pro-inflammatory cytokines such as TNF, IL-1β, and IL-6 (46). This process is further complicated by oxidative stress and nitric oxide (NO)-mediated neurotoxicity. This study found that, following tACS intervention, the expression of multiple pro-inflammatory molecules (including iNOS, IL-1β, IL-6, COX-2 and M1 microglia markers) showed an overall significant downregulation trend, while reparative phenotype indicators (such as Arg-1 and other M2-associated markers) were relatively upregulated. These results imply that tACS could correct the imbalance immune microenvironment after SCI by modulating the polarization of microglia, shifting it from a pro-inflammatory and neurotoxic state to an anti-inflammatory and repair-promoting state. This immune phenotype shift aligns closely with the reduced tissue damage and improved motor function observed in histological staining, neuronal survival quantification and behavioral functional tests (e.g. BBB scores and gait analysis). These results demonstrate that the immune microenvironment remodeling induced by tACS is reflected not only at the molecular level but also translates into substantial benefits in terms of neural structure protection and functional recovery.

### 4.2 The cGMP-PKG pathway is a key molecular hub that regulates the microglial phenotype via tACS

Proteomics analysis in this study revealed that proteins that were differentially expressed following tACS intervention were significantly enriched in key functional modules, such as the ‘cGMP-PKG signaling pathway’. Combined with ELISA-detected elevated cGMP levels and Western blot validation of PKG protein expression, this further confirms the effective activation of this pathway under the influence of tACS. As a classic second messenger system, the cGMP-PKG pathway exerts multifaceted effects in neuroprotection and immunoregulation (47, 48). On the one hand, it suppresses the release of proinflammatory mediators such as TNF, IL-1β and IL-6 by modulating the activity of inflammation-related transcription factors such as NF-κB and their downstream signaling cascades (34). Conversely, this pathway creates a more favorable ‘mild’ internal environment for tissue repair and neuronal survival by improving local microcirculation, reducing oxidative stress damage and enhancing cell survival signals such as Akt and CREB (49, 50).

In pharmacologic mechanism validation experiments, the introduction of the specific PKG inhibitor KT5823 was found to significantly attenuate or partially reverse tACS-induced anti-inflammatory effects (e.g. downregulation of proinflammatory factors and upregulation of the M2 phenotype) and neuroprotective actions (e.g. enhanced neuronal survival and improved axonal integrity). These results strongly suggest that the cGMP-PKG pathway is not merely a passive by-product of tACS intervention, but rather a critical driving mechanism that underpins its immunomodulatory and neuroprotective functions. Consequently, this study marks a pivotal transition in elucidating the underlying mechanisms, elevating our findings from mere correlational associations derived from omics data to robust causal validation achieved via pharmacological blockade. This deepens our understanding of tACS’s mechanism of action and substantially enhances the logical rigor and scientific credibility of the research conclusions.

### 4.3 How tACS ‘connects’ central nervous activity to immune phenotypes: Potential pathways for neuroimmune coupling

tACS noninvasively modulates cortical oscillatory rhythms and neural network excitability by applying weak currents at specific frequencies (51). Its therapeutic benefits in cases of SCI likely stem from two mechanisms: ‘top-down neural circuit regulation’ and ‘indirect anti-inflammatory effects mediated through neuro-immune coupling’. On the one hand, tACS enhances activity in cortical-spinal projection pathways, thereby improving the functional plasticity of motor-related neural networks. This promotes axonal sprouting, regeneration and synaptic reconstruction. Significant enrichment of synaptic function-related pathways, such as ‘synaptic vesicle trafficking’, in the proteomics data from this study provides molecular-level support for this theory. This suggests that tACS may lay the neural foundation for long-term motor recovery by reshaping the efficiency and structural integrity of synaptic transmission. Conversely, tACS may exert effects by modulating autonomic nervous system tone, influencing neurotransmitter release (e.g. norepinephrine, acetylcholine and 5-HT), improving local energy metabolism and reshaping the glial microenvironment. These mechanisms may reduce the sensitivity of microglia to damage-associated molecular patterns (DAMPs), increase their response thresholds and encourage a shift from the ‘killer/cleaner’ (M1-like) phenotype to the ‘repairer/homeostatic maintainer’ (M2-like) phenotype.

The cascade reaction prominently features the cGMP-PKG signaling pathway: Alterations in neuronal firing patterns initiate intracellular second messenger cascades, which subsequently govern transcriptional programs associated with inflammation. It is highly likely to serve as the ‘molecular interface’ that converts external electrical stimulation signals into intrinsic immune phenotype remodeling. Further research into the upstream triggering mechanisms of tACS activation in this pathway could propel tACS beyond its status as a phenomenologically effective intervention towards a new era of precision neuromodulation characterized by clear mechanisms, defined targets and optimizable parameters. This research could investigate whether tACS activation involves NO-sGC axis activation, phosphodiesterase (PDE) inhibition or signaling mediated by specific neurotransmitter receptors (e.g. β-adrenergic receptors or 5-HT receptors).

### 4.4 Potential mechanism linking cortex to spinal cord: tACS may indirectly activate the cGMP-PKG pathway via remote neuromodulation

The precise mechanism by which tACS translates into biological signals within the injured spinal cord is still unknown. While our data confirms that the cGMP-PKG pathway is a critical downstream mediator, several potential upstream mechanisms could still bridge the ‘cortical-to-spinal cord’ signaling gap.

One such mechanism is the modulation of descending neural pathways. It is well established that tACS can modulate cortical oscillations and alter the excitability of large-scale neural networks (18, 52). Even following SCI, residual corticospinal tracts and other descending fibers remain intact, and tACS may enhance the activity of these pathways. This would directly influence the electrophysiological state and neuroimmune function of spinal cord cells. Our proteomics data reveal significant enrichment of the ‘synaptic vesicle recycling’ pathway, which provides strong molecular-level support for this hypothesis.

Secondly, vascular-related mechanisms may also play a significant role in this process. The cGMP-PKG pathway is a classic vasodilatory signaling axis that plays a central role in regulating vascular smooth muscle relaxation and local tissue perfusion (53, 54). Therefore, tACS may improve the local blood supply and microcirculation in the injured spinal cord by activating relevant neurovascular regulatory mechanisms. This creates a more favorable microenvironment for tissue repair, metabolic recovery, and inflammation resolution.

In summary, tACS is unlikely to act on the injured spinal cord via a single pathway; rather, its effects are likely to be the result of the synergistic involvement of multiple processes, including the regulation of neural activity, neurovascular coupling, and the remodeling of the immune microenvironment.

### 4.5 Cellular experimental results support the translatable inflammatory mediators–neuronal injury mechanism

In an *in vitro* model in which LPS-activated BV2 microglia were co-cultured with VSC4.1 neuron-like cells, we observed that modulating the inflammatory state of the microglia had a significant influence on the survival and death outcomes of the downstream neurons (as demonstrated by Live/Dead staining). This finding provides more direct causal evidence for the ‘decreased inflammation–enhanced neuronal protection–improved motor function’ association observed in *in vivo* studies. Specifically, this vitro system clearly demonstrates that the neuroprotective effect of tACS is not merely manifested as behavioral compensation or symptom relief. Rather, it substantially remodels the secretum and the overall inflammatory burden of microglia, thereby reducing their paracrine toxic stress on neighboring neurons and mitigating inflammatory neuronal injury. This neuron-protective effect, driven by improvements in the immune microenvironment, was independently validated *in vitro*, eliminating interference from systemic or circuit-level confounding factors.

Taken together, the convergence of *in vitro* and *in vivo* evidence validates the mechanistic chain linking glial polarization to functional recovery via neuronal survival. This comprehensive approach allows us to move beyond phenomenological description, offering definitive causal evidence that significantly strengthens the persuasiveness of this study.

### 4.6 Clinical translation implications and study limitations

From a translational medicine perspective, tACS shows promise as a key component of comprehensive SCI rehabilitation strategies due to its non-invasive nature, reproducibility and adjustable parameters. Validating its immunomodulatory effects in larger animal models and in cases of diverse injury types could make it particularly suitable for cases involving pronounced secondary inflammatory responses requiring long-term intervention. Furthermore, given that the cGMP-PKG signaling pathway is pharmacologically modifiable, combining electrical stimulation with pathway enhancement could have synergistic effects. However, this study has several limitations:

Firstly, comprehensive dose-response and frequency-specific analysis of tACS parameters (frequency, intensity and intervention time window) are incomplete. This necessitates further exploration of optimal treatment protocols and individual variability. Secondly, the M1/M2 classification is based on an oversimplified model of microglial activation states. The microglial lineage exhibits greater continuity and heterogeneity. Future studies should therefore integrate single-cell transcriptomics or spatial omics technologies to delineate these complex phenotypic clusters more precisely. Furthermore, while the cGMP-PKG pathway has been shown to play a vital role in tACS-mediated neuroprotection, the most promising intervention points require further clarification of key downstream targets and upstream triggering mechanisms. Finally, as this study was based solely on one species, sex and experimental model, its findings are not widely generalizable. Subsequent research should validate the stability and reliability of these effects across different injury severities, sex differences and chronic-phase models.

Secondly, the limitations inherent in the classical M1/M2 dichotomy employed in this study must be acknowledged. Although this binary classification provides a useful framework for interpreting general inflammatory trends, it necessarily oversimplifies the complex and continuous spectrum of microglial activation states. Microglia exhibit profound heterogeneity and context-dependent plasticity that may not be fully captured by canonical markers alone. Consequently, future investigations should incorporate single-cell transcriptomics or spatial omics technologies to delineate these complex phenotypic clusters more precisely.

Furthermore, while our data highlights the modulation of microglia, this should be interpreted as a significant contributor to the observed neuroprotection, rather than the primary established mechanism. As a neuromodulator intervention, tACS is likely to influence a diverse array of cell types within the injured spinal cord. For example, tACS could directly affect neuronal excitability and synaptic plasticity by modulating neural oscillations (18). The therapeutic benefits observed are likely the result of a synergistic interplay among neurons, astrocytes, oligodendrocytes and microglia (20). Therefore, while the polarization of microglia is a pivotal factor, the relative contributions of these other neural and glial populations warrant further investigation to fully elucidate the comprehensive mechanism of tACS.

### 4.7 Limitations regarding the relationship between *in vitro* and *in vivo* mechanisms

In summary, while tACS shows great promise for treating SCI, overcoming the challenges is essential before it can be used clinically. More diverse research designs and accumulated data are needed to provide robust scientific evidence for personalized precision medicine.

Objectively speaking, it should be noted that it is currently impossible to establish a perfect direct correlation between *in vitro* and *in vivo* experimental results. In the *in vitro* studies, we used the pharmacological activator 8-Br-cGMP-AM to simulate the downstream effects of the cGMP-PKG pathway. In contrast, the *in vivo* experiments used tACS without applying direct electrical stimulation to the cultured BV2 microglial cells.

This experimental design was based primarily on considerations regarding the mechanism of action of tACS. As a complex neuromodulation technique, tACS’s effects often depend on ‘top-down’ neural circuit regulation (e.g. the corticospinal tract) and neuroimmune coupling mechanisms rather than direct stimulation of isolated immune cells. After all, the electric field distribution and physiological environment within a culture dish cannot fully replicate the complex regulatory environment in which tACS operates *in vivo*.

Consequently, while *in vitro* data confirms that the cGMP-PKG pathway has the molecular capacity to regulate microglial polarization and *in vivo* data indicates that tACS effects depend on this pathway, the full mechanism by which tACS-mediated changes in microglial membrane potential occur via the cGMP-PKG pathway requires further validation in a simplified cellular system. Future research will introduce microfluidic devices to more accurately simulate the *in vivo* electrophysiological microenvironment, enabling a more in-depth investigation of the direct sensitivity of microglia to tACS.

### 4.8 Limitations regarding electric field distribution and mechanism

Although our results demonstrate the efficacy of tACS in modulating the T10 spinal cord microenvironment, the physical mechanism of this modulation requires further investigation. In the frontoparietal–ventral thoracic electrode configuration employed in this study, the electric field distribution is anticipated to be more widespread than strictly focal. Due to their complex anatomical structure and distinct conductive properties, tissues such as the skull, cerebrospinal fluid, vertebrae, muscle and thoracoabdominal organs cannot be ignored. Consequently, the electric field intensity reaching the T10 injury site is likely to be significantly weaker than in the cortical region.

Therefore, the neuroprotective effects observed in the spinal cord are more likely to be the result of network- and system-level regulation, such as the modulation of descending corticospinal pathways and neuro-immune coupling, rather than being the direct result of the injury site receiving sufficiently strong local electric field stimulation. Our proteomics data support this hypothesis, showing significant enrichment in the ‘synaptic vesicle cycle’ pathway and indicating altered neural network activity. Future studies should combine high-resolution image reconstruction with finite element modeling and intersegmental electrophysiological recordings to delineate stimulation pathways more accurately and define the effective range of action within the injured spinal cord.

### 4.9 Limitations of the pharmacological inhibitor KT5823

In this study, we used the phosphodiesterase type 1 inhibitor KT5823 (3 mg/kg/day, administered intraperitoneally) to validate the role of the cGMP-PKG pathway in mediating the effects of tACS. While this approach provides key causal evidence, several limitations of this pharmacological tool must be acknowledged. Firstly, KT5823 is known to have potential off-target effects on other kinases, such as PKA and PKC, particularly at higher concentrations. While the dosage used in this study was chosen based on prior research to specifically target PKG in the central nervous system, it is not possible to eliminate the possibility that the inhibition of other kinases may have contributed to the observed outcomes. Secondly, as KT5823 is a systemically administered agent, it inhibits PKG activity throughout the body. Given the key role of the cGMP-PKG pathway in regulating cardiovascular function, platelet aggregation, and smooth muscle tone, systemic inhibition may induce physiological changes, such as alterations in spinal cord perfusion, which could influence the prognosis of SCI independently. Therefore, while the reversal of tACS-mediated benefits by KT5823 strongly suggests the involvement of the cGMP-PKG pathway, these systemic effects may also confound the results. Future studies utilizing more specific inhibitors or conditional, cell-type-specific PKG knockout animal models will be crucial for elucidating the role of PKG in the spinal cord microenvironment following tACS intervention.

## 5 Conclusion

In summary, this study provides compelling evidence that tACS effectively suppresses the polarization of microglia towards an M1-like proinflammatory phenotype following SCI, thereby activating the cGMP-PKG signaling pathway. This significantly reduces the neuroinflammatory burden, thereby protecting neurons from secondary damage and ultimately promoting tissue repair and the recovery of motor function (**Figure 10**). This discovery provides clearer and deeper immunological support for the application of tACS in the treatment of SCI, and, for the first time, organically links exogenous electrical stimulation with endogenous second messenger pathways and immune microenvironment remodeling. It outlines a potential mechanistic cascade wherein electrical stimulation activates the cGMP-PKG pathway to modulate glial phenotypes, ultimately contributing to neuroprotection and functional recovery. Furthermore, this research establishes a solid theoretical basis for the development of “electro-pharmacological” intervention strategies that though the cGMP-PKG pathway. This approach has the potential to advance SCI rehabilitation, shifting the focus from single-modality physical regulation to a new phase characterized by multimodal, precision-driven, and mechanism-based approaches.

**Figure 10:**
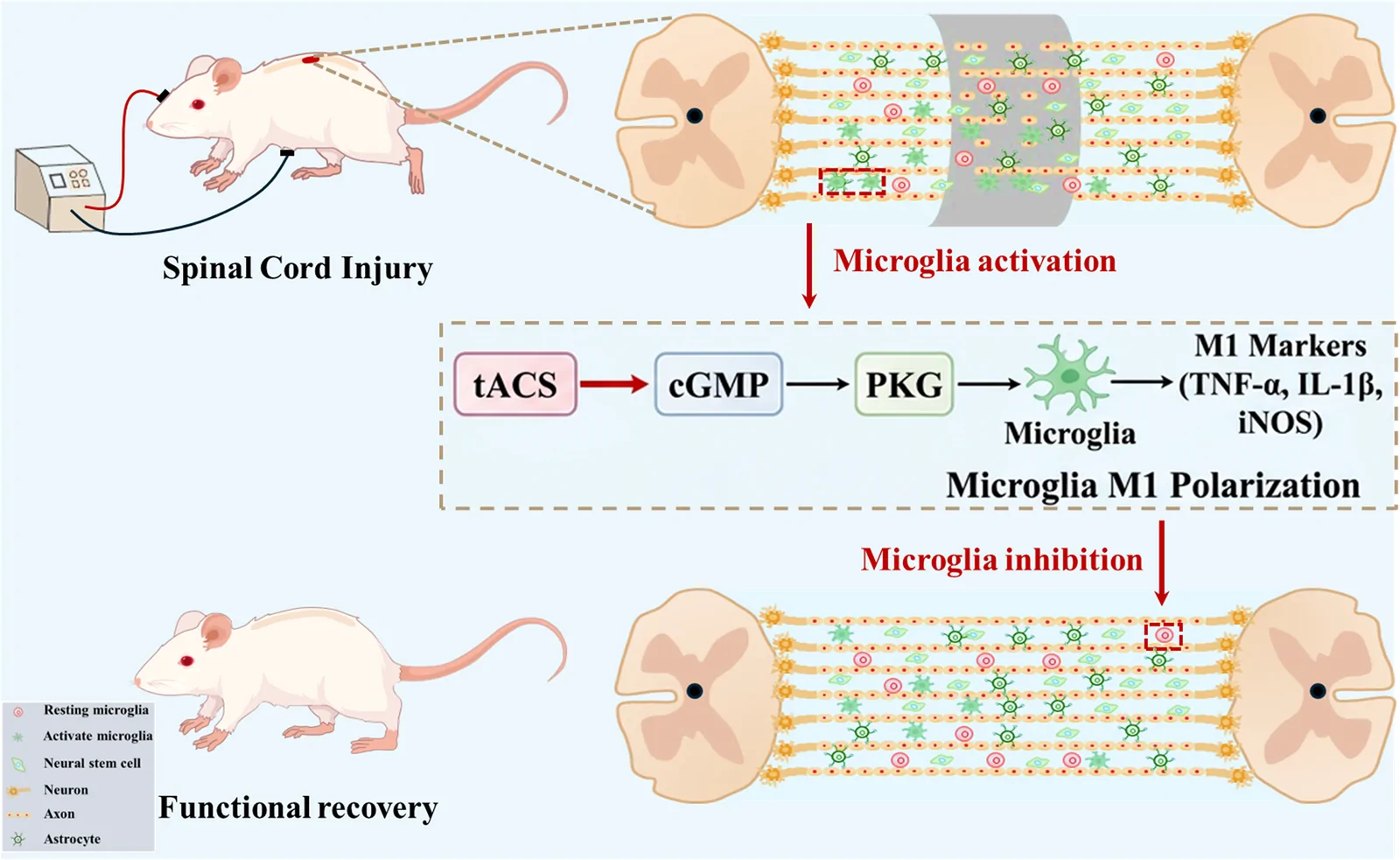
Schematic diagram illustrating the mechanism by which tACS promotes motor function recovery after SCI by stabilizing the cGMP-PKG pathway, thereby inhibiting microglial polarization toward the M1 phenotype.

## Data Availability

The original contributions presented in the study are included in the article/**Supplementary Material**. Further inquiries can be directed to the corresponding authors.
