# Supplementary material for: tACS suppresses M1 polarization of microglia after spinal cord injury through the cGMP-PKG signaling pathway
Source: Front Immunol. 2026 Oct 7;17:1907391. doi: 10.3389/fimmu.2026.1907391 (PMC13683643; doi:10.3389/fimmu.2026.1907391)
Supplement: Supplementary file 1 [file Table1.docx]

**S1** Quantitative PCR primers.
